# The association between hypertriglyceridemic-waist phenotype and chronic kidney disease: a cohort study and meta-analysis

**DOI:** 10.1038/s41598-022-05806-7

**Published:** 2022-02-04

**Authors:** Dezhong Chen, Huimin Sun, Ciyong Lu, Weiqing Chen, Vivian Yawei Guo

**Affiliations:** Department of Epidemiology, School of Public Health, Sun Yat-Sen University, 74 Zhongshan Second Road, Guangzhou, 510080 Guangdong China

**Keywords:** Metabolic disorders, Chronic kidney disease

## Abstract

Evidence on the association between hypertriglyceridemic-waist phenotype (HTGW) and chronic kidney disease (CKD) is limited and inconsistent. We aimed to explore such association among 7406 Chinese aged ≥ 45 years in a cohort setting, followed by a meta-analysis. Participants were categorized into four phenotypes: NTNW (normal triglycerides and normal waist circumference), NTGW (isolated enlarged waist circumference), HTNW (isolated high triglycerides), and HTGW (high triglycerides and enlarged waist circumference). We used multivariate logistic regression to determine the association between different phenotypes and risk of CKD in the cohort study. For meta-analysis, we searched relevant studies from Embase, Medline, PubMed, and Web of Science from dataset inception up to May 1, 2021. A random-effect model was used to estimate the pooled effect and *I*^2^ statistic was applied to evaluate heterogeneity. In the cohort study, compared to the NTNW phenotype, HTGW (OR 1.82, 95% CI 1.32 to 2.51, *p* < 0.01) and NTGW (OR 1.48, 95% CI 1.13 to 1.94, *p* = 0.004) were significantly associated with CKD risk after 4 years follow-up, but not for the HTNW phenotype. The meta-analysis also showed a positive association between HTGW phenotype and CKD risk (pooled OR 1.53, 95% CI 1.31 to 1.79, *I*^2^ = 62.4%). Assessment of triglyceridemic-waist phenotypes might help to identify individuals with high-risk of developing CKD.

## Introduction

Chronic kidney disease (CKD) has become a major public health concern worldwide because of its increasing prevalence and devastating complications^1^. Globally, the estimated prevalence of CKD has reached 9.1% with 697.5 million recorded cases in 2017^1^. In China, a nationwide survey conducted in 2009 showed that approximately 10.8% of the adults were suffering from CKD, imposing a significant burden to the health system^2^. CKD was not only the leading cause of end stage renal disease^3^, but also associated with increased risk of cardiovascular disease (CVD)^4^. The risk of mortality was substantially elevated in patients with CKD as well^5^.

Several risk factors have been linked to the risk of CKD, such as advanced age, genetics, diabetes mellitus (DM), and hypertension^6^. Central obesity, a state with excess accumulation of fat in the abdominal area, is also associated with increased risk of CKD^7–10^. A cohort study conducted in the US showed that participants with larger waist circumference had increased risk of CKD^7^. A meta-analysis of 21 cohort studies further confirmed that elevated waist circumference was associated with higher risk of declines in estimated glomerular filtration rate (eGFR), an indicator of kidney function that bears great importance in clinical decision-making^10^. In addition, evidence has also suggested a link between high triglyceride levels and CKD risk^11–13^. For example, the triglyceride levels were observed to be positively associated with incident CKD in a cohort study of 15,244 participants in China^12^. Similarly, another retrospective study enrolled 10,288 participants suggested that high triglycerides was an independent risk factor for CKD^13^.

Due to the close link of central obesity and high triglyceride levels with the risk of CKD, hypertriglyceridemic-waist phenotype (HTGW), i.e. simultaneous presence of central obesity and elevated triglyceride levels, was also found to be associated with renal impairment^14–23^. A cross-sectional study with 1,828 adults aged between 18 and 75 years has found that the risk of impaired renal function was significantly higher in participants with HTGW compared to those with normal waist circumference and normal triglyceride levels^14^. In another study conducted among 12,012 individuals from Iran, HTGW was associated with 37% increased likelihood of having CKD^19^. However, inconsistent findings were also reported. A cross-sectional study in China showed that the significant association between HTGW and CKD was only present in males, but not in females^17^. In contrast, another prospective analysis found that HTGW was not significantly associated with CKD, regardless of the sex^19^. A cross-sectional study further demonstrated that HTGW was linked to mildly and moderately reduced eGFR (eGFR between 30 and 89 mL/min/1.73 m^2^), but not severely reduced eGFR (eGFR between 15 and 29 mL/min/1.73 m^2^)^16^.

These inconsistent findings suggested the needs for further investigations. Furthermore, most of current studies were cross-sectional in nature^14–18,20–23^, which cannot determine the temporal association between the exposure and the outcome. Therefore, we aimed to investigate whether HTGW phenotype was associated with the development of CKD based on the China health and retirement longitudinal study (CHARLS). Stratified analyses were further conducted to identify potential modifying variables. Additionally, we included the results in a meta-analysis with those from prior studies.

## Methods

### Study design and population

**Figure 1:**
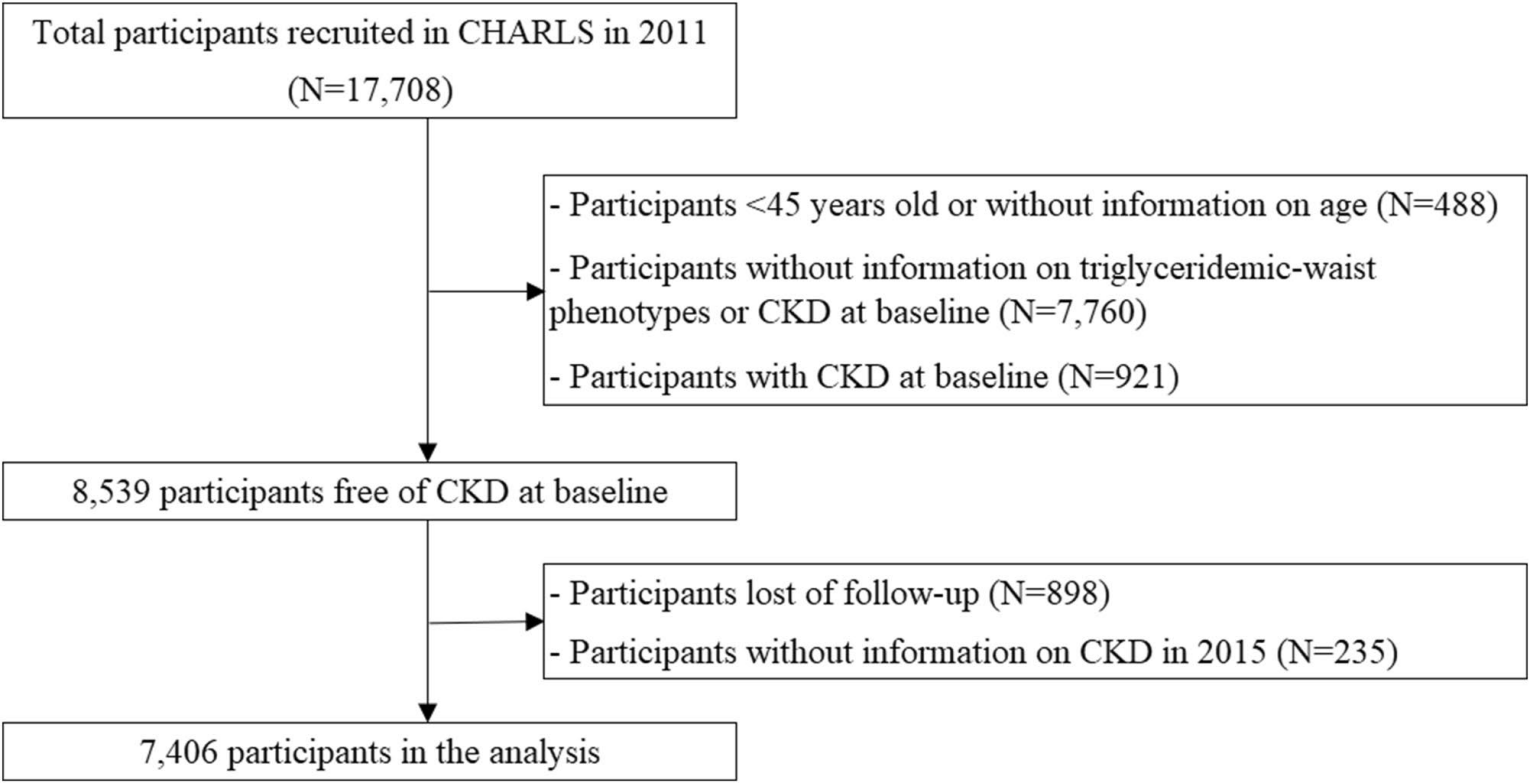
Flow chart of participants selection in CHARLS.

CHARLS is a nationally representative study comprised of participants aged 45 years old and above. The details of the study have been described previously^24,25^. In brief, multistage probability sampling strategy was applied to select participants at baseline. The final study population were drawn from 450 villages/urban communities in 150 counties/districts of 28 provinces across China. Every two years, participants were followed up again with a small share of new respondents. We used data collected from baseline and the 2015 follow-up survey, as blood samples were assessed during these two surveys. Between June 2011 and March 2012, a total of 17,708 participants from 10,287 households were interviewed during the baseline assessment (Fig. 1). We excluded participants aged < 45 years old or without age information (*n* = 488), participants without information on triglyceridemic-waist phenotypes or CKD (*n* = 7760), and those with CKD diagnosis at baseline (*n* = 921). Of the remaining 8539 participants who were free of CKD at baseline, we further excluded participants that were lost of follow-up (*n* = 898) or without information on CKD diagnosis in the 2015 survey (*n* = 235). Finally, 7406 eligible participants were included in the current analysis.

All participants provided signed informed consent at the time of participation. Ethical approval for the CHARLS was granted by the Institutional Review Board at Peking University. The Institutional Review Board approval number was IRB00001052-11015 for the main household survey and IRB00001052-11014 for the biomarker collection.

### Exposure definition

Waist circumference was measured with a soft tape at the level of the navel when participants were asked to hold their breath at the end of exhale. Enlarged waist circumference, i.e., central obesity, was defined as a waist circumference ≥ 90 cm in men and ≥ 85 cm in women^26^. Triglycerides was measured by enzymatic colorimetric test^27^. A triglyceride level ≥ 1.7 mmol/L was considered abnormal^28^. Participants were divided into four phenotypes according to their triglycerides levels and waist circumference^29^: (1) normal triglyceride levels and normal waist circumference (NTNW); (2) normal triglyceride levels with enlarged waist circumference (NTGW); (3) high triglyceride levels and normal waist circumference (HTNW); (4) high triglyceride levels and enlarged waist circumference (HTGW).

### Outcome definition

Serum creatinine was evaluated by the Rate-blanked and compensated Jaffe creatinine method. We used the Chronic Kidney Disease Epidemiology Collaboration equation to calculated eGFR as follows^30^: (1) female: eGFR = 141 × min (serum creatinine/61.9, 1)^-0.329^ × max (serum creatinine/61.9, 1)^-1.209^ × 0.993^age^ × 1.018; (2) male: eGFR = 141 × min (serum creatinine/79.6, 1)^-0.411^ × max (serum creatinine/79.6, 1)^-1.209^ × 0.993^age^. The units for eGFR and serum creatinine were mL/min/1.73 m^2^ and μmol/L, respectively. The “min” in the equation means the minimum value between serum creatinine/61.9 and 1 in female, or serum creatinine/79.6 and 1 in male. The “max” in the equation means the maximum value between serum creatinine/61.9 and 1 in female, or serum creatinine/79.6 and 1 in male. CKD was defined as self-reported physician diagnosed CKD and/or eGFR < 60 mL/min/1.73 m^2^.

### Measurement of covariates

Sociodemographic characteristics and lifestyle factors were collected by trained investigators through face-to-face interviews. The residence of participants was classified into urban and rural areas. Education level was grouped into illiterate or without formal education, primary school, middle school, and high school or above. Smoking and drinking status were divided into current users *versus* non-current users. A small part of the participants (42.4%) additionally answered questions about their physical activities. Sufficient physical activity was defined as having at least 75 min of vigorous physical activity, or 150 min of moderate physical activity, or 600 metabolic equivalent value of moderate-to-vigorous physical activity each week according to the World Health Organization guidelines^31^. Body mass index (BMI) was calculated as weight (kg) divided by height squared (m^2^). Overweight was defined as BMI ≥ 24 kg/m^2^ according to the recommended standard for Chinese adults^32^. Blood pressure was measured three times with at least 45 s intervals using a digital sphygmomanometer (Omron TM HEM-7200 Monitor, Japan) and the average values were used in the analysis. Total cholesterol, high-density lipoprotein cholesterol (HDL-c), and low-density lipoprotein cholesterol (LDL-c) were measured by enzymatic colorimetric test^27^. High-sensitivity C-reactive protein was measured with immunoturbidimetric assay. According to the American Diabetes Association criteria^33^, a participant was defined as having DM if any of the following criteria was met: (1) fasting plasma glucose ≥ 7.0 mmol/L; (2) random plasma glucose ≥ 11.1 mmol/L; (3) glycated hemoglobin (HbA1c) ≥ 6.5%; (4) self-reported physician diagnosed DM; and (5) on hypoglycemic agents including glucose-lowering drugs and insulin. Hypertension was defined if any of the following criteria was met: (1) mean systolic blood pressure (SBP) ≥ 140 mmHg; (2) mean diastolic blood pressure (DBP) ≥ 90 mmHg; (3) self-reported physician diagnosed hypertension; and (4) on anti-hypertensive agents. CVD was defined as having self-report of physician diagnosed stroke and/or coronary heart disease.

### Search strategy and selection criteria for meta-analysis

The meta-analysis was conducted according to the recommendations of the Preferred Reporting Items for Systematic Review and Meta-Analysis (PRISMA) guidelines^34^. The protocol of current meta-analysis has been registered in PROSPERO (CRD42021284912). In brief, Embase, PubMed, Medline, and Web of Science were searched from dataset inception up to May 1, 2021 for papers that have assessed the association between HTGW phenotype and risk of CKD. The search strategy included combination of terms related to HTGW and CKD without any restrictions on language or article type (details in Supplementary Methods). The references of the included studies and relevant reviews were also searched for any potential papers. Grey literatures were further searched from the US National Technical Information Service (http://www.ntis.gov/) and Open Grey (http://www.opengrey.eu/). After removing duplicated papers, irrelevant ones were excluded through screening titles and abstracts. Of the remaining papers, full text was reviewed for further selection.

Original observational studies that have estimated the association between HTGW and CKD were included in the meta-analysis. Case reports, case series, experimental models, meta-analyses, reviews, responses, and letters were excluded. A standardized form was used to record information of authors, publication year, country, study design, sample size, percentage of males, mean age, cut-off values of suboptimal waist circumference and triglycerides, outcome definition, adjusted covariates, and risk estimates of the association. Two reviewers (DC and HS) independently searched and screened papers, and extracted data from the identified studies. Disagreements were resolved through discussion.

### Statistical analysis

#### Cohort study

Descriptive statistics were expressed as mean ± standard deviation (SD) for continuous variables with normal distribution, or median (interquartile range) for continuous variables with skewed distribution. Categorical variables were expressed as frequency (percentage). The differences of baseline characteristics across four triglyceridemic-waist phenotypes were compared by one-way analysis of variance (ANOVA) for continuous variables with normal distribution, or Kruskal–Wallis test for continuous variables with skewed distribution. Categorical variables were compared by chi-square test. We further compared the baseline characteristics between participants developed CKD during the follow-up period and those without. In this comparison, independent student’s t test and Wilcoxon rank-sum test were used for comparison of continuous variables with normal or skewed distribution, respectively. Chi-square test was used for categorical variables between the two groups.

We used multivariate logistic regression analyses to determine the association between different triglyceridemic-waist phenotypes and the risk of CKD, with normal triglyceride levels and normal waist circumference group (i.e. NTNW) as the reference. Model 1 only included the triglyceridemic-waist phenotypes, without adjustment for any covariate. Model 2 adjusted for baseline age, sex, residence, education level, smoking and drinking status, BMI, HDL-c, history of DM, hypertension, and CVD, use of hypoglycemic agents, anti-hypertensive agents, and lipid-regulating agents, as well as baseline eGFR levels. Model 3 additionally adjusted for physical activity. These adjusting variables were selected based on empirical evidence and availability in CHARLS^6,12,35–37^. The results of multivariate logistic regression analyses were presented as odds ratio (OR) with corresponding 95% confidence interval (CI). Stratified analysis was conducted according to age (< 60 or ≥ 60 years), sex (male or female), BMI status (< 24 kg/m^2^ or ≥ 24 kg/m^2^), drinking status (current users or non-current users), history of DM (yes or no), and history of hypertension (yes or no).

#### Meta-analysis

A pooled OR was used to assess the association between HTGW phenotype and risk of CKD in the meta-analysis. *I*^2^ statistic was applied to test the heterogeneity between included studies and a *p* value less than 0.05 was considered to be statistically significant^38^. A random-effect model with inverse variance was used if the preliminary combination indicated significant heterogeneity; otherwise, a fixed-effect model was applied. The quality of included studies was assessed by Newcastle–Ottawa quality scale^39^. A leave-one-out sensitivity analysis by omitting one study at a time and re-calculating the summarized risk estimates was also conducted to assess the impact of each study on the pooled risk estimates. Publication bias was first visually inspected through funnel plot. Begg’s and Egger’s tests were further performed to assess the publication bias and a *p* value less than 0.05 indicated significant publication bias^40,41^. Subgroup analysis was used to assess the sex-specific association.

Data analyses were conducted using STATA 15.0 (StataCorp. College Station, TX, USA) and metafor package from R software. Two-tailed *P* values < 0.05 were considered as statistically significant.

## Results

### Baseline characteristics according to the triglyceridemic-waist phenotypes

Table 1 shows the baseline characteristics of participants according to different triglyceridemic-waist phenotypes. Of the 7406 participants without CKD at baseline, the prevalence of NTNW, NTGW, HTNW, and HTGW were 47.2%, 26.6%, 10.1%, and 16.0%, respectively. In general, compared to participants with normal triglyceride levels and normal waist circumference, those with HTGW phenotype were more likely to be urban residents, non-current smokers or drinkers, and had higher SBP and DBP, higher prevalence of DM, hypertension, and CVD. Additionally, the mean eGFR was the lowest in the HTGW group at baseline.

**Table 1:** Comparison of baseline characteristics according to different triglyceridemic-waist phenotypes.

	NTNW	NTGW	HTNW	HTGW	*P* value
*N* (%)	3495 (47.2%)	1972 (26.6%)	751 (10.1%)	1188 (16.0%)	
**Sociodemographic and lifestyle factors**					
Sex, *n* (%)					< 0.001
Male	1941 (55.5%)	635 (32.2%)	398 (53.0%)	382 (32.2%)	
Female	1554 (44.5%)	1337 (67.8%)	353 (47.0%)	806 (67.8%)	
Age, years	59.5 ± 9.1	59.0 ± 9.0	58.0 ± 8.7	58.5 ± 8.5	< 0.001
Ethnicity, *n* (%)					0.587
Han ethnicity	3170 (93.4%)	1774 (92.9%)	670 (93.3%)	1090 (94.2%)	
Other minorities	223 (6.6%)	135 (7.1%)	48 (6.7%)	67 (5.8%)	
Residence, *n* (%)					< 0.001
Rural	2530 (72.4%)	1206 (61.2%)	525 (69.9%)	689 (58.0%)	
Urban	965 (27.6%)	766 (38.8%)	226 (30.1%)	499 (42.0%)	
Education level, *n* (%)					0.112
Illiterate or without formal education	1724 (49.3%)	967 (49.1%)	339 (45.1%)	547 (46.0%)	
Primary school	804 (23.0%)	414 (21.0%)	177 (23.6%)	279 (23.5%)	
Middle school	640 (18.3%)	408 (20.7%)	165 (22.0%)	243 (20.5%)	
High school or above	326 (9.3%)	182 (9.2%)	70 (9.3%)	119 (10.0%)	
Current smoker, *n* (%)	1320 (37.9%)	374 (19.0%)	277 (37.0%)	231 (19.5%)	< 0.001
Current alcohol user, *n* (%)	1338 (38.3%)	499 (25.3%)	264 (35.2%)	323 (27.2%)	< 0.001
Physical activity, *n* (%)^†^					< 0.001
Insufficient	411 (28.4%)	336 (37.8%)	93 (31.3%)	213 (42.0%)	
Sufficient	1037 (71.6%)	552 (62.2%)	204 (68.7%)	294 (58.0%)	
**Clinical/biochemical measures**					
BMI (kg/m^2^)	21.3 ± 2.7	26.0 ± 3.3	22.2 ± 2.5	26.9 ± 3.2	< 0.001
**Waist circumference (cm)**					
Male	79.5 ± 5.9	96.2 ± 5.4	81.7 ± 5.8	97.4 ± 5.6	< 0.001
Female	76.7 ± 5.5	92.8 ± 6.5	78.6 ± 4.6	94.6 ± 6.9	< 0.001
SBP (mmHg)	126.1 ± 20.5	134.1 ± 21.4	128.6 ± 20.1	136.5 ± 21.3	< 0.001
DBP (mmHg)	73.1 ± 11.7	78.0 ± 11.9	75.2 ± 11.8	79.7 ± 12.1	< 0.001
Plasma glucose (mmol/L)	5.8 ± 1.3	6.0 ± 1.9	6.5 ± 2.3	7.1 ± 2.8	< 0.001
Total cholesterol (mmol/L)	4.8 ± 0.9	5.0 ± 0.9	5.3 ± 1.0	5.4 ± 1.1	< 0.001
Triglycerides (mmol/L)	0.9 (0.7–1.2)	1.1 (0.9–1.4)	2.3 (1.9–2.9)	2.4 (2.0–3.2)	< 0.001
HDL-c (mmol/L)	1.5 ± 0.4	1.3 ± 0.3	1.1 ± 0.3	1.0 ± 0.3	< 0.001
LDL-c (mmol/L)	2.9 ± 0.8	3.2 ± 0.8	2.9 ± 1.1	3.0 ± 1.1	< 0.001
Serum creatinine (μmol/L)	67.7 ± 13.9	65.4 ± 13.6	69.0 ± 14.7	67.2 ± 14.4	< 0.001
C-reactive protein (mg/L)	0.8 (0.5–1.7)	1.2 (0.6–2.3)	1.0 (0.5–2.0)	1.4 (0.8–2.8)	< 0.001
eGFR (ml/min/1.73m^2^)	94.0 ± 12.2	93.2 ± 12.6	93.5 ± 13.8	91.5 ± 13.5	< 0.001
History of chronic diseases					
DM, *n* (%)	316 (9.0%)	278 (14.1%)	137 (18.2%)	339 (28.5%)	< 0.001
Hypertension, *n* (%)	1009 (28.9%)	973 (49.3%)	279 (37.2%)	699 (58.8%)	< 0.001
CVD, *n* (%)	339 (9.7%)	278 (14.1%)	77 (10.3%)	219 (18.5%)	< 0.001
**Medications**					
Hypoglycemic agents, *n* (%)	53 (1.5%)	80 (4.1%)	23 (3.1%)	81 (6.8%)	< 0.001
Anti-hypertensive agents, *n* (%)	342 (9.8%)	492 (24.9%)	102 (13.6%)	371 (31.2%)	< 0.001
Lipid-regulating agents, *n* (%)	66 (1.9%)	119 (6.0%)	27 (3.6%)	114 (9.6%)	< 0.001

*NTNW* normal triglyceride levels and normal waist circumference, *NTGW* normal triglyceride levels with enlarged waist circumference, *HTNW* high triglyceride levels and normal waist circumference, *HTGW* high triglyceride levels and enlarged waist circumference, *BMI* body mass index, *SBP* systolic blood pressure, *DBP* diastolic blood pressure, *HDL-c* high-density lipoprotein cholesterol, *LDL-c* low-density lipoprotein cholesterol, *eGFR* estimated glomerular filtration rate, *DM* diabetes mellitus, *CVD* cardiovascular disease.

^†^ Physical activity was only available for 3,140 participants.

### Associations of triglyceridemic-waist phenotypes with CKD

After 4 years of follow-up, 580 (7.8%) participants developed CKD. Compared to participants who did not develop CKD, those developed CKD were older, less educated, and more likely to have insufficient physical activity (Supplementary Table S1). Incident CKD cases also tended to have larger waist circumference in females, higher triglycerides and serum creatinine levels, lower eGFR, and higher prevalence of DM, hypertension, and CVD, compared to their non-CKD counterparts at baseline.

The cumulative incidence rates of CKD over 4 years were 6.6%, 8.4%, 7.3%, and 10.9% in NTNW, NTGW, HTNW, and HTGW groups, respectively. Table 2 presents the associations between triglyceridemic-waist phenotypes and the risk of CKD. Compared to the NTNW phenotype, NTGW (OR 1.30, 95% CI 1.06 to 1.61) and HTGW (OR 1.73, 95% CI 1.38 to 2.17) groups had significantly higher risks of CKD in the crude model. The trends were still significant with adjustment of covariates in model 2 and model 3. In contrast, compared to the NTNW group, the risk of CKD was not significantly increased in the group with isolated higher triglyceride levels at follow-up in any of the model.

**Table 2:** Associations between triglyceridemic-waist phenotypes and incident chronic kidney disease.

	NTNW	NTGW		HTNW		HTGW	
	OR (95% CI)	OR (95% CI)	*P* value	OR (95% CI)	*P* value	OR (95% CI)	*P* value
Model 1	1.00 (ref)	1.30 (1.06–1.61)	0.012	1.12 (0.83–1.52)	0.461	1.73 (1.38–2.17)	< 0.001
Model 2	1.00 (ref)	1.48 (1.13–1.94)	0.004	1.16 (0.83–1.63)	0.367	1.82 (1.32–2.51)	< 0.001
Model 3	1.00 (ref)	1.96 (1.31–2.94)	0.001	1.44 (0.85–2.42)	0.175	2.53 (1.56–4.10)	< 0.001

*NTNW* normal triglyceride levels and normal waist circumference, *NTGW* normal triglyceride levels with enlarged waist circumference, *HTNW* high triglyceride levels and normal waist circumference, *HTGW* high triglyceride levels and enlarged waist circumference, *OR* odds ratio, *CI* confidence interval.

Model 1 only included the triglyceridemic-waist phenotypes without adjustment for any covariate. Model 2 adjusted for baseline age, sex, residence, education level, smoking and drinking status, BMI, HDL-c, history of DM, hypertension, and CVD, use of hypoglycemic agents, anti-hypertensive agents, and lipid-regulating agents, as well as baseline eGFR levels. Model 3 additionally adjusted for physical activity, with only 3,100 participants having available data.

### Stratified analysis

Compared to NTNW group, the risk of CKD for HTGW phenotype remained statistically significant among both younger and older adults, females, participants with normal weight, non-current drinkers, and those without DM or hypertension at baseline (Table 3). For the NTGW group, its significant association with CKD was present in all stratified groups, except for participants aged ≥ 60 years, participants with overweight, participants who were currently drinkers, or those with a history of DM. No significant associations were found between HTNW group and the risk of CKD in any of the stratified groups, except for non-current drinkers. We further found that current drinking status was as significant effect modifier in the association between triglyceridemic-waist phenotypes and risk of CKD, while age, sex, BMI, history of DM or hypertension were not.

**Table 3:** Stratified analysis of the associations between triglyceridemic-waist phenotypes and incident chronic kidney disease.

Variables	*N*	Case, *n* (%)	NTNW	NTGW		HTNW		HTGW		*P* for interaction
			OR (95% CI)	OR (95% CI)	*P* value	OR (95% CI)	*P* value	OR (95% CI)	*P* value	
**Age**										
< 60 years	4106	233 (5.7%)	1.00 (ref)	1.55 (1.02–2.34)	0.038	1.11 (0.66–1.86)	0.689	1.99 (1.22–3.24)	0.006	0.680
≥ 60 years	3300	347 (10.5%)	1.00 (ref)	1.41 (0.98–2.02)	0.061	1.19 (0.76–1.86)	0.445	1.69 (1.09–2.61)	0.019	
**Sex**										
Male	3356	283 (8.4%)	1.00 (ref)	1.57 (1.05–2.36)	0.030	1.13 (0.73–1.76)	0.581	1.53 (0.91–2.56)	0.106	0.319
Female	4050	297 (7.3%)	1.00 (ref)	1.45 (1.01–2.10)	0.046	1.25 (0.75–2.10)	0.396	2.15 (1.39–3.32)	0.001	
**BMI**										
< 24 kg/m^2^	4304	313 (7.3%)	1.00 (ref)	1.63 (1.10–2.37)	0.012	1.21 (0.83–1.76)	0.314	2.30 (1.38–3.83)	0.001	0.301
≥ 24 kg/m^2^	3102	267 (8.6%)	1.00 (ref)	1.13 (0.72–1.78)	0.585	0.85 (0.38–1.90)	0.689	1.38 (0.84–2.27)	0.208	
**Drinking status**										
Current users	2424	198 (8.2%)	1.00 (ref)	1.60 (0.99–2.57)	0.053	0.67 (0.35–1.28)	0.227	1.38 (0.75–2.52)	0.297	0.009
Non-current users	4973	382 (7.7%)	1.00 (ref)	1.51 (1.08–2.10)	0.016	1.56 (1.05–2.33)	0.029	2.11 (1.42–3.12)	< 0.001	
**History of diabetes mellitus**										
Yes	1070	108 (10.1%)	1.00 (ref)	1.07 (0.52–2.22)	0.838	1.01 (0.45–2.24)	0.988	1.78 (0.84–3.78)	0.131	0.772
No	6336	472 (7.5%)	1.00 (ref)	1.57 (1.17–2.10)	0.002	1.20(0.83–1.74)	0.336	1.77 (1.22–2.55)	0.002	
**History of hypertension**										
Yes	2960	266 (9.0%)	1.00 (ref)	1.55 (1.02–2.34)	0.039	1.26 (0.75–2.14)	0.385	1.60 (0.99–2.58)	0.056	0.752
No	4446	314 (7.1%)	1.00 (ref)	1.45 (1.01–2.09)	0.044	1.13 (0.72–1.75)	0.597	2.08 (1.32–3.27)	0.002	

*NTNW* normal triglyceride levels and normal waist circumference, *NTGW* normal triglyceride levels with enlarged waist circumference, *HTNW* high triglyceride levels and normal waist circumference, *HTGW* high triglyceride levels and enlarged waist circumference, *OR* odds ratio, *CI* confidence interval.

Except for the stratified variables in each stratum, analysis adjusted for baseline age, sex, residence, education level, smoking and drinking status, BMI, HDL-c, history of DM, hypertension and CVD, use of hypoglycemic agents, anti-hypertensive agents, and lipid-regulating agents, as well as baseline eGFR levels.

### Meta-analysis

**Figure 2:**
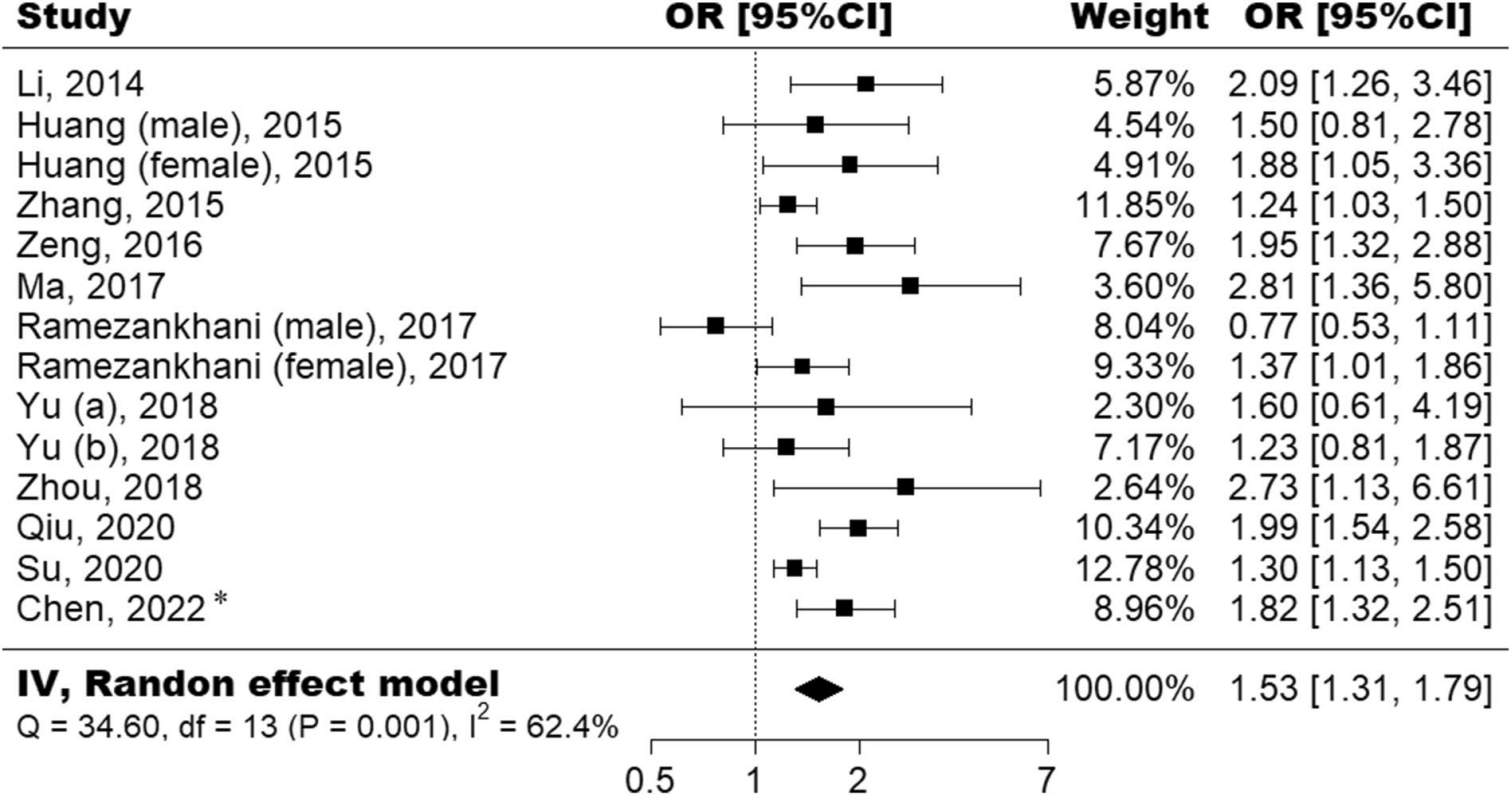
Forest plot summarizing the association between hypertriglyceridemic-waist phenotype and chronic kidney disease. ^*^Data from current study.

Supplementary Fig. S1 illustrates the study selection process in the meta-analysis. Finally, ten published studies met the inclusion criteria and were included in the present meta-analysis with our cohort analysis^14–23^. The details of each study were summarized in Supplementary Table S2 and S3. Figure 2 shows the study-specific ORs and the pooled risk estimates. HTGW was positively associated with CKD risk (OR 1.53, 95% CI 1.31 to 1.79) with significant heterogeneity (*I*^2^ = 62.4%, *p* = 0.001). The leave-one-out analysis showed that omitting any study would not alter the trend or significance of the pooled estimates (Supplementary Fig. S2). Supplementary Fig. S3 presents the funnel plot. Both Begg’s test (*p* = 0.412) and Egger’s test (*p* = 0.120) indicated that there was no evidence of potential publication bias. Subgroup analysis showed that the pooled risk estimates were significant in both males (OR 1.38, 95% CI 1.04 to 1.82) and females (OR 1.66, 95% CI 1.31 to 2.10) (Supplementary Figs. S4 and S5).

## Discussion

In this cohort study of Chinese aged 45 years and above, both NTGW and HTGW phenotypes were linked to increased risk of CKD compared to the NTNW phenotype, whereas HTNW was not significantly associated with incident CKD over a 4-year follow-up period. The findings were generally consistent in participants at different ages, females, participants with normal weight, non-current drinkers, individuals without DM, and those without hypertension. Current drinking status was a significant effect modifier in the association between HTGW and incident CKD. The meta-analysis also revealed a positively significant association between HTGW phenotype and CKD risk.

The current finding on the increased risk of CKD among HTGW individuals were consistent with several previous studies^15,16,18,20,21,23^. For example, a cross-sectional study conducted in China showed that participants with HTGW phenotype were more likely to have CKD (OR 2.09, 95% CI 1.26 to 3.45)^15^. In another study with relatively lean participants (BMI < 24 kg/m^2^) from southern China also revealed similar associations after adjusting for confounders (OR 2.73, 95% CI 1.13 to 6.62)^18^. In terms of the associations between NTGW and HTNW groups with CKD risk, only the NTGW phenotype showed a significantly elevated CKD risk compared to participants with NTNW in our present study. The results were similar to a cross-sectional study of 31,296 adults aged 20–74 years in China^16^. Nevertheless, inconsistent findings were also observed^17,19^. For example, a cross-sectional study incorporating 2102 urban elderly aged over 60 years showed that the association between triglyceridemic-waist phenotypes and CKD risk was only significant among participants with HTGW phenotype, but not for those with NTGW or HTNW phenotypes^17^. Another prospective Iranian study even showed that none of the triglyceridemic-waist phenotypes was significantly associated with the risk of CKD^19^.

One possible explanation for the inconsistency across relevant studies might be the different study designs. Unlike most previous studies that applied a cross-sectional analysis^14–18,20–23^, our study used longitudinal data, which was more powerful to determine the temporal association between the exposure and the outcome. Also, enlarged waist circumference in women and high triglycerides levels were defined differently across published research. Some studies used a cut-off of 80 cm to define central obesity in women^14,16,17,23^. Instead, we used 85 cm, which was considered an appropriate cut-off in Chinese women for central obesity^26^. Similarly, several studies used 2.0 mmol/L to define high triglycerides^15,18,19,21,22^, while we used the cut-off of 1.7 mmol/ L, which was recommended to define metabolic syndrome in Chinese^28^. Also, previous research has shown that triglyceride levels above 1.7 mmol/L was already significantly associated with increased risk of CKD^42^. Additionally, several studies combined NTGW and HTNW phenotypes into one group in the analysis^14,15,18^, while we examined the individual impact of each triglyceridemic-waist phenotype on the risk of CKD. Furthermore, in the multivariate logistic regression models, we additionally adjusted medications for treating DM, hypertension, and dyslipidemia, which have been demonstrated to be associated with the risk of CKD^36^. Therefore, the differences we observed across studies were plausible. These differences across studies might also have caused the significant heterogeneity observed in the meta-analysis.

The mechanisms underlying the association between HTGW and CKD remain incompletely elucidated. HTGW has been considered a marker of visceral obesity^43^. Evidence has suggested that visceral adiposity, rather than subcutaneous adiposity, was more closely related to metabolic abnormalities, such as insulin resistance, hypertension, and dyslipidemia^44^, which were all well-established risk factors of CKD^45^. Furthermore, previous research has demonstrated that increased inflammation played an important role in the pathogenesis of renal disease^46^. In our study, the HTGW group had the highest levels of C-reactive protein, a marker of systematic inflammation. The increased level of inflammation might further cause tubulointerstitial damage, and eventually the development of CKD^47^. In addition, previous research has suggested a link between HTGW phenotype and reduced level of adiponectin, an anti-inflammatory adipokine^48^. Lower adiponectin levels could reduce the activation of 5'-AMP-activated protein kinase (AMPK) in podocytes and further lead to albuminuria and CKD^49^. Therefore, the significant associations we observed between HTGW and CKD is biologically plausible.

Although not statistically significant, stratified analyses suggested that the risk estimates on the association between HTGW and CKD risk in females were relatively higher than that in males, which have been consistently reported in previous studies^14,19^. Our study only included adults aged 45 years and above. Most of the female participants at this age were experiencing menopause with declined levels of estrogen, a hormone that has protective effects on renal function^50^. Declined estrogen levels during menopause period might attenuate its protective effect on renal function, and lead to increased vulnerability of CKD development in women^51^. This was also supported by a patient-level meta-analysis, which showed that the rate of progression to renal disease was relatively faster in post-menopausal women than men^52^. The gender-specific analysis in the meta-analysis also revealed reduced risk estimates in men, compared to women. However, the association was statistically significant in men as well, which might be due to the wide age range of the study populations that were included in the meta-analysis. In addition, we only found the significant associations between HTGW and CKD in normal-weight participants, but not in those with overweight or obesity. Among individuals with overweight or obesity, adipose tissues were enlarged with an increased secretion of pro-inflammatory adipokines and cytokines^53,54^. The increased inflammation could subsequently impair renal function and increase the risk of CKD^55^, regardless of their triglyceridemic-waist phenotypes.

In the stratified analysis by drinking status, the consumption of alcohol was a significant effect modifier in the association between HTGW and CKD risk, with higher risk estimates in non-current drinkers. Previous research has shown that heavy drinking and alcohol dependence may increase the risk of CKD^56,57^. Therefore, in current drinkers, the associations between HTGW and CKD might be attenuated by alcohol-induced rhabdomyolysis, hypertension, and its related toxic effects^58–60^. Nevertheless, a recent meta-analysis found that alcohol consumption was associated with reduced risk of CKD^61^. The discrepancy might be caused by the different polyphenol contents in wine, beer, and gin, which could exert different impact on kidney^62,63^. Future studies are needed to further elucidate the modification effect of alcohol consumption with consideration of its types, quantity, and duration. In addition, the significant associations between HTGW and CKD seemed only evident in participants without history of DM and hypertension. Previous studies have shown that DM and hypertension were independent risk factors for CKD even after adjustment for central obesity and triglycerides^6^. Therefore, in patients with DM or hypertension, the risk of CKD might have already elevated, no matter whether they had HTGW phenotype or not. However, the finding was in contrary to a cross-sectional study published previously^16^. Further studies are needed to confirm the associations.

Our study has several strengths, including the prospective design, various stratified analyses and adjustment for several established confounders. Some limitations should also be addressed. First, although participants were selected from different districts in China using a multistage probability sampling method, a large proportion was excluded due to missing data or loss of follow-up. Therefore, the generalizability of our findings might be limited. Second, as urine samples were not collected in the CHARLS surveys, we were unable to define CKD based on albuminuria, which might potentially underestimate the number of CKD cases. Third, evidence has demonstrated a link between CKD and some potential risk factors, such as dietary intake^64^ and sedentary time^65^. However, due to the lack of relevant information, we were unable to adjust for these factors or exclude the possibility of residual confounders. Thus, the associations between HTGW phenotype and risk of CKD might be overestimated in our current analysis.

## Conclusions

In conclusion, our results showed that in Chinese adults aged 45 years and above, participants with HTGW or isolated high waist circumference were at increased risk of developing CKD compared to those with normal triglyceridemic levels and normal waist circumference. Meta-analysis also confirmed the findings. Our study suggested that simultaneous measurement of triglyceride and waist circumference might be a useful screening tool to identify high-risk individuals of developing CKD. However, further studies are needed to evaluate the generalizability and cost-effectiveness of such a tool.

## Supplementary Information

Supplementary Information.

## Data Availability

The cohort data underlying this article are available in a public, open access repository, and can be accessed at website of CHARLS http://charls.pku.edu.cn/index/en.html (access on 15 September 2020).
