# Supplementary material for: The association between hypertriglyceridemic-waist phenotype and chronic kidney disease: a cohort study and meta-analysis
Source: Sci Rep. 2022 Feb 4;12:1935. doi: 10.1038/s41598-022-05806-7 (PMC8817025; doi:10.1038/s41598-022-05806-7)
Supplement: Supplementary file 1 — Supplementary Information. [file 41598_2022_5806_MOESM1_ESM.docx]

**Online Supplemental Material**

**PRISMA checklist**

**Supplementary Methods** | **Search terms in each database**

**Supplementary Table S1** | **Comparison of baseline characteristics by CKD status at follow-up**

**Supplementary Table S2** | **Characteristics of the studies included in meta-analysis**

**Supplementary Table S3** | **Summary of covariates and risk estimates of the studies included in meta-analysis**

**Supplementary Fig S1** | **PRISMA flow diagram of the study selection process**

**Supplementary Fig S2** | **Examining the influence of individual study by leave-one-out method**

**Supplementary Fig S3** | **Funnel plot of included studies**

**Supplementary Fig S4** | **Forest plot summarizing the association between hypertriglyceridemic-waist phenotype and chronic kidney disease in males**

**Supplementary Fig S5** | **Forest plot summarizing the association between hypertriglyceridemic-waist phenotype and chronic kidney disease in females**

**PRISMA checklist**

| **Section and Topic** | **Item #** | **Checklist item** | **Location where item is reported** |
| --- | --- | --- | --- |
| **TITLE** | | |  |
| Title | 1 | Identify the report as a systematic review. | 1 |
| **ABSTRACT** | | |  |
| Abstract | 2 | See the PRISMA 2020 for Abstracts checklist. | 2 |
| **INTRODUCTION** | | |  |
| Rationale | 3 | Describe the rationale for the review in the context of existing knowledge. | 3-4 |
| Objectives | 4 | Provide an explicit statement of the objective(s) or question(s) the review addresses. | 4-5 |
| **METHODS** | | |  |
| Eligibility criteria | 5 | Specify the inclusion and exclusion criteria for the review and how studies were grouped for the syntheses. | 8-9 |
| Information sources | 6 | Specify all databases, registers, websites, organisations, reference lists and other sources searched or consulted to identify studies. Specify the date when each source was last searched or consulted. | 8 |
| Search strategy | 7 | Present the full search strategies for all databases, registers and websites, including any filters and limits used. | Supplementary Methods |
| Selection process | 8 | Specify the methods used to decide whether a study met the inclusion criteria of the review, including how many reviewers screened each record and each report retrieved, whether they worked independently, and if applicable, details of automation tools used in the process. | 9 |
| Data collection process | 9 | Specify the methods used to collect data from reports, including how many reviewers collected data from each report, whether they worked independently, any processes for obtaining or confirming data from study investigators, and if applicable, details of automation tools used in the process. | 9 |
| Data items | 10a | List and define all outcomes for which data were sought. Specify whether all results that were compatible with each outcome domain in each study were sought (e.g. for all measures, time points, analyses), and if not, the methods used to decide which results to collect. | Supplementary Table S2 and S3 |
|  | 10b | List and define all other variables for which data were sought (e.g. participant and intervention characteristics, funding sources). Describe any assumptions made about any missing or unclear information. | Supplementary Table S2 and S3 |
| Study risk of bias assessment | 11 | Specify the methods used to assess risk of bias in the included studies, including details of the tool(s) used, how many reviewers assessed each study and whether they worked independently, and if applicable, details of automation tools used in the process. | 10-11 |
| Effect measures | 12 | Specify for each outcome the effect measure(s) (e.g. risk ratio, mean difference) used in the synthesis or presentation of results. | 10-11 |
| Synthesis methods | 13a | Describe the processes used to decide which studies were eligible for each synthesis (e.g. tabulating the study intervention characteristics and comparing against the planned groups for each synthesis (item #5)). | NA |
|  | 13b | Describe any methods required to prepare the data for presentation or synthesis, such as handling of missing summary statistics, or data conversions. | 10-11 |
|  | 13c | Describe any methods used to tabulate or visually display results of individual studies and syntheses. | 10-11 |
|  | 13d | Describe any methods used to synthesize results and provide a rationale for the choice(s). If meta-analysis was performed, describe the model(s), method(s) to identify the presence and extent of statistical heterogeneity, and software package(s) used. | 10-11 |
|  | 13e | Describe any methods used to explore possible causes of heterogeneity among study results (e.g. subgroup analysis, meta-regression). | 10-11 |
|  | 13f | Describe any sensitivity analyses conducted to assess robustness of the synthesized results. | 10-11 |
| Reporting bias assessment | 14 | Describe any methods used to assess risk of bias due to missing results in a synthesis (arising from reporting biases). | 10-11 |
| Certainty assessment | 15 | Describe any methods used to assess certainty (or confidence) in the body of evidence for an outcome. | NA |
| **RESULTS** | | |  |
| Study selection | 16a | Describe the results of the search and selection process, from the number of records identified in the search to the number of studies included in the review, ideally using a flow diagram. | Supplementary Fig S1 |
|  | 16b | Cite studies that might appear to meet the inclusion criteria, but which were excluded, and explain why they were excluded. | NA |
| Study characteristics | 17 | Cite each included study and present its characteristics. | Supplementary Table S2 and S3 |
| Risk of bias in studies | 18 | Present assessments of risk of bias for each included study. | Supplementary Table S2 |
| Results of individual studies | 19 | For all outcomes, present, for each study: (a) summary statistics for each group (where appropriate) and (b) an effect estimate and its precision (e.g. confidence/credible interval), ideally using structured tables or plots. | Fig 2 |
| Results of syntheses | 20a | For each synthesis, briefly summarise the characteristics and risk of bias among contributing studies. | NA |
|  | 20b | Present results of all statistical syntheses conducted. If meta-analysis was done, present for each the summary estimate and its precision (e.g. confidence/credible interval) and measures of statistical heterogeneity. If comparing groups, describe the direction of the effect. | Fig 2 |
|  | 20c | Present results of all investigations of possible causes of heterogeneity among study results. | Supplementary Fig S4 and S5 |
|  | 20d | Present results of all sensitivity analyses conducted to assess the robustness of the synthesized results. | Supplementary Fig S2 |
| Reporting biases | 21 | Present assessments of risk of bias due to missing results (arising from reporting biases) for each synthesis assessed. | 13 |
| Certainty of evidence | 22 | Present assessments of certainty (or confidence) in the body of evidence for each outcome assessed. | NA |
| **DISCUSSION** | | |  |
| Discussion | 23a | Provide a general interpretation of the results in the context of other evidence. | 14-15 |
|  | 23b | Discuss any limitations of the evidence included in the review. | 17 |
|  | 23c | Discuss any limitations of the review processes used. | 17 |
|  | 23d | Discuss implications of the results for practice, policy, and future research. | 19 |
| **OTHER INFORMATION** | | |  |
| Registration and protocol | 24a | Provide registration information for the review, including register name and registration number, or state that the review was not registered. | 8 |
|  | 24b | Indicate where the review protocol can be accessed, or state that a protocol was not prepared. | 8 |
|  | 24c | Describe and explain any amendments to information provided at registration or in the protocol. | NA |
| Support | 25 | Describe sources of financial or non-financial support for the review, and the role of the funders or sponsors in the review. | 19 |
| Competing interests | 26 | Declare any competing interests of review authors. | 19 |
| Availability of data, code and other materials | 27 | Report which of the following are publicly available and where they can be found: template data collection forms; data extracted from included studies; data used for all analyses; analytic code; any other materials used in the review. | 20 |

**Supplementary Methods. Search terms in each database**

Embase, PubMed, Medline, and Web of Science were searched from dataset inception up to May 1, 2021

(i) Original observational studies that estimated the association between hypertriglyceridemic-waist phenotypes and CKD or related biomarkers were included in the meta-analysis.

(ii) Case reports, case series, experimental models, meta-analyses, reviews, responses, and letters were excluded.

**PubMed**

**Search terms:**

1. “Hypertriglyceridemic Waist”[Mesh] or hypertriglyceridemic waist or hypertriglyceridaemic waist or hypertriglyceridemic waist phenotype or hypertriglyceridaemic waist phenotype or HTGW or HWHT or EWHT or ((enlarged waist) and (high triglyceride*)) or HTW or HW

2. “renal insufficiency, chronic”[MeSH] or CKD or chronic kidney disease or kidney disease or kidney failure or chronic kidney failure or renal failure or chronic kidney disorder or kidney disorder or chronic renal disease or CKF or CRF or CRD or decreased eGFR or reduced eGFR or renal impairment or creatinine or albuminuria or urine albumin or proteinuria or urine protein or end stage kidney disease or end stage renal disease or kidney function or chronic nephropathy

3. 1 and 2

**Embase**

**Search terms:**

1. 'hypertriglyceridemic waist'/exp OR 'hypertriglyceridemic waist'

2. hypertriglyceridaemic AND ('waist'/exp OR waist)

3. htgw

4. hwht

5. ewht

6. enlarged AND ('waist'/exp OR waist) AND high AND ('triglyceride'/exp OR triglyceride)

7. htw

8. hw

9. ckd

10. 'chronic kidney failure'/exp OR 'chronic kidney failure'

11. 'kidney failure'/exp OR 'kidney failure'

12. 'kidney disease'/exp OR 'kidney disease'

13. ('kidney'/exp OR kidney) AND ('disorder'/exp OR disorder)

14. ckf

15. 'crf'/exp OR crf

16. crd

17. decreased AND ('egfr'/exp OR egfr)

18. reduced AND ('egfr'/exp OR egfr)

19. 'renal impairment'/exp OR 'renal impairment'

20. 'creatinine'/exp OR 'creatinine'

21. 'albuminuria'/exp OR 'albuminuria'

22. 'proteinuria'/exp OR 'proteinuria'

23. 'protein urine level'/exp OR 'protein urine level'

24. 'end stage renal disease'/exp OR 'end stage renal disease'

25. 'kidney function'/exp OR 'kidney function'

26. chronic AND ('nephropathy'/exp OR nephropathy)

27. 1 or 2 or 3 or 4 or 5 or 6 or 7or 8

28. or 9 or 10 or 11 or 12 or 13 or 14 or 15 or 16 or 17 or 18 or 19 or 20 or 21 or 22 or 23 or 24 or 25 or 26

29. 27 and 28

**OVID Medline**

**Search terms:**

1. Hypertriglyceridemic Waist.mp. or exp Hypertriglyceridemic Waist/

2. (enlarged waist and high triglyceride*).mp. [mp=title, abstract, original title, name of substance word, subject heading word, floating sub-heading word, keyword heading word, organism supplementary concept word, protocol supplementary concept word, rare disease supplementary concept word, unique identifier, synonyms]

3. hypertriglyceridaemic waist.mp.

4. exp Hypertriglyceridemic Waist/ or HTGW.mp.

5. exp Hypertriglyceridemic Waist/ or EWHT.mp.

6. HTW.mp. or exp Hypertriglyceridemic Waist/

7. HW.mp.

8. 1 or 2 or 3 or 4 or 5 or 6 or 7

9. kidney disease.mp. or exp Kidney Diseases/

10. kidney failure.mp. or exp Renal Insufficiency/

11. exp Kidney Diseases/ or kidney disorder.mp. or exp Kidney/

12. kidney insufficiency.mp. or exp Renal Insufficiency/

13. exp Renal Insufficiency, Chronic/ or exp Kidney Failure, Chronic/ or renal disease.mp. or exp Kidney Diseases/ or exp Kidney/

14. renal failure.mp. or exp Renal Insufficiency/

15. exp Kidney Failure, Chronic/ or exp Kidney Diseases/ or exp Kidney/ or renal disorder.mp.

16. renal insufficiency.mp. or exp Renal Insufficiency/

17. exp Kidney Failure, Chronic/ or CKF.mp. or exp Kidney Diseases/

18. CRF.mp.

19. CRD.mp.

20. exp Glomerular Filtration Rate/ or exp Renal Insufficiency, Chronic/ or exp Kidney Failure, Chronic/ or CKD.mp. or exp Kidney/ or exp Kidney Diseases/

21. exp Renal Insufficiency, Chronic/ or decreased eGFR.mp. or exp Kidney/ or exp Glomerular Filtration Rate/

22. exp Albuminuria/ or exp Glomerular Filtration Rate/ or exp Renal Insufficiency, Chronic/ or reduced eGFR.mp. or exp Kidney Diseases/

23. exp Kidney/ or exp Kidney Diseases/ or renal impairment.mp. or exp Renal Insufficiency/

24. creatinine.mp. or exp Kidney/ or exp Creatinine/ or exp Kidney Failure, Chronic/

25. albuminuria.mp. or exp Albuminuria/

26. exp Albuminuria/ or exp Creatinine/ or urine albumin.mp.

27. exp Proteinuria/ or proteinuria.mp.

28. exp Kidney Diseases/ or exp Renal Insufficiency, Chronic/ or exp Proteinuria/ or urine protein.mp. or exp Creatinine/ or exp Kidney/

29. end stage kidney disease.mp. or exp Kidney Failure, Chronic/

30. end stage renal disease.mp. or exp Kidney Failure, Chronic/

31. exp Renal Insufficiency, Chronic/ or exp Kidney Diseases/ or kidney function.mp.

32. exp Kidney Diseases/ or exp Kidney Failure, Chronic/ or chronic nephropathy.mp. or exp Renal Insufficiency, Chronic/

33. 9 or 10 or 11 or 12 or 13 or 14 or 15 or 16 or 17 or 18 or 19 or 20 or 21 or 22 or 23 or 24 or 25 or 26 or 27 or 29 or 30 or 31 or 32

34. 8 and 33

**Web of Science**

**Search terms:**

1. (hypertriglyceridemic waist) or (hypertriglyceridaemic waist) or (hypertriglyceridemic waist phenotype) or (hypertriglyceridaemic waist phenotype) or (hypertriglyceridaemic waist phenotype) or (HTGW) or (HWHT) or (EWHT) or "enlarged waist and high triglyceride*" or (HTW) or (HW)

2. (chronic kidney disease) or (kidney disease) or (chronic kidney failure) or (kidney failure) or (chronic kidney disorder) or (kidney disorder) or (chronic kidney insufficiency) or (kidney insufficiency) or (chronic renal disease) or (renal disease) or (chronic renal failure) or (renal failure) or (chronic renal disorder) or (renal disorder) or (chronic renal insufficiency) or (renal insufficiency) or (CKF) or (CRF) or (CRD) or (CKD) or (decreased eGFR) or (reduced eGFR) or (renal impairment) or (creatinine) or (albuminuria) or (urine albumin) or (proteinuria) or (urine protein) or (end stage kidney disease) or (end stage renal disease) or (kidney function) or (chronic nephropathy)

3. 1 and 2

**Supplementary Table S1. Comparison of baseline characteristics by CKD status at follow-up**

|  | | **CKD** | **Non-CKD** | **P value** |
| --- | --- | --- | --- | --- |
| **N (%)** | | 580 (7.8%) | 6,826 (92.2%) |  |
| **Sociodemographic and lifestyle factors** | |  |  |  |
| Sex, n (%) | |  |  | 0.080 |
|  | Male | 283 (48.8%) | 3,073 (45.0%) |  |
|  | Female | 297 (51.2%) | 3,753 (55.0%) |  |
| Age, years | | 62.2±9.2 | 58.8±8.9 | <0.001 |
| Ethnicity, n (%) | |  |  | 0.568 |
|  | Han ethnicity | 531 (94.0%) | 6,173 (93.4%) |  |
|  | Other minorities | 34 (6.0%) | 439 (6.6%) |  |
| Residence, n (%) | |  |  | 0.178 |
|  | Rural | 373 (64.3%) | 4,577 (67.1%) |  |
|  | Urban | 207 (35.7%) | 2,249 (32.9%) |  |
| Education level, n (%) | |  |  | 0.005 |
|  | Illiterate or without formal education | 301 (52.0%) | 3,276 (48.0%) |  |
|  | Primary school | 147 (25.4%) | 1,527 (22.4%) |  |
|  | Middle school | 87 (15.0%) | 1,369 (20.1%) |  |
|  | High school or above | 44 (7.6%) | 653 (9.6%) |  |
| Current smoker, n (%) | | 166 (28.9%) | 2,036 (29.9%) | 0.246 |
| Current alcohol user, n (%) | | 198 (34.1%) | 2,226 (32.7%) | 0.535 |
| Physical activity, n (%) | |  |  | 0.019 |
|  | Insufficient | 105 (40.1%) | 948 (32.9%) |  |
|  | Sufficient | 157 (59.9%) | 1,930 (67.1%) |  |
| **Clinical / biochemical measures** | |  |  |  |
| BMI (kg/m²) | | 23.8±3.7 | 23.6±3.8 | 0.204 |
| Waist circumference (cm) | |  |  |  |
|  | Male | 86.0±10.3 | 84.9±9.6 | 0.078 |
|  | Female | 88.0±10.4 | 85.6±10.2 | <0.001 |
| SBP (mmHg) | | 132.8±23.0 | 129.9±21.1 | 0.004 |
| DBP (mmHg) | | 75.9±12.7 | 75.6±12.1 | 0.627 |
| Plasma glucose (mmol/L) | | 6.3±2.5 | 6.1±1.9 | 0.142 |
| Total cholesterol (mmol/L) | | 5.1±1.0 | 5.0±1.0 | 0.204 |
| Triglycerides (mmol/L) | | 1.3 (0.9-1.9) | 1.2 (0.8-1.7) | <0.001 |
| HDL-c (mmol/L) | | 1.29±0.41 | 1.33±0.39 | 0.036 |
| LDL-c (mmol/L) | | 3.0±1.0 | 3.0±0.9 | 0.636 |
| Serum creatinine (μmol/L) | | 72.5±15.6 | 66.7±13.8 | <0.001 |
| C-reactive protein (mg/L) | | 1.1 (0.7-2.3) | 1.0 (0.5-2.1) | <0.001 |
| eGFR (ml/min/1.73m²) | | 86.8±14.3 | 93.9±12.4 | <0.001 |
| **History of chronic diseases** | |  |  |  |
| DM, n (%) | | 108 (18.6%) | 962 (14.1%) | 0.003 |
| Hypertension, n (%) | | 266 (45.9%) | 2,694 (39.5%) | 0.003 |
| CVD, n (%) | | 114 (19.7%) | 799 (11.7%) | <0.001 |
| **Medications** | |  |  |  |
| Hypoglycemic agents, n (%) | | 31 (5.3%) | 206 (3.0%) | 0.002 |
| Anti-hypertensive agents, n (%) | | 138 (23.8%) | 1,169 (17.1%) | <0.001 |
| Lipid-regulating agents, n (%) | | 49 (8.4%) | 277 (4.1%) | <0.001 |

CKD, chronic kidney disease; BMI, body mass index; SBP, systolic blood pressure; DBP, diastolic blood pressure; HDL-c, high-density lipoprotein cholesterol; LDL-c, low-density lipoprotein cholesterol; eGFR, estimated glomerular filtration rate; DM, diabetes mellitus; CVD, cardiovascular disease.

**Supplementary Table S2. Characteristics of the studies included in meta-analysis**

| **Author, year** | **Country** | **Study design** | **Sample size** | **Male (%)** | **Age (years)** | **WC cut-off (cm)** | | **TG cut-off (mmol/L)** | | **Disease outcomes** |
| --- | --- | --- | --- | --- | --- | --- | --- | --- | --- | --- |
|  |  |  |  |  |  | **M** | **F** | **M** | **F** |  |
| Li, 2014 ^1^ | China | Cross-sectional study | 1,534 | 37.0 | 57.21±10.97 | 90 | 85 | 2.0 | 2.0 | CKD was defined as an eGFR of less than 60 ml/min per 1.73 m^2^ or albuminuria (urinary albumin-to-creatinine ratio higher than 30 mg/g). |
| Huang, 2015 ^2^ | China | Cross-sectional study | 1,828 | 37.4 | Total: 18-75  Male: 53.14±15.03  Female: 52.3±14.19 | 90 | 80 | 1.7 | 1.7 | CKD was defined as an eGFR of less than 60 ml/min per 1.73 m^2^ or albuminuria (urinary albumin-to-creatinine ratio higher than 30 mg/g). |
| Zhang, 2015 ^3^ | China | Cross-sectional study | 2,502 | 46.0 | 56.1 | 85 | 80 | 1.7 | 1.7 | Microalbuminuria (30-300 mg/24h). |
| Zeng, 2016 ^4^ | China | Cross-sectional study | 2,102 | 40.3 | 71.2±6.6 | 90 | 80 | 1.7 | 1.7 | CKD was defined as an eGFR of less than 60 ml/min per 1.73 m^2^ or albuminuria (urinary albumin-to-creatinine ratio higher than 30 mg/g). |
| Ma, 2017 ^5^ | China | Cross-sectional study | 538 | 50.1 | >18 | 90 | 85 | 1.7 | 1.7 | Early diabetic nephropathy was defined as urine microalbumin among 20-200 μg/min. |

**Supplementary Table S2. Characteristics of the studies included in meta-analysis (continue)**

| **Author, year** | **Country** | **Study design** | **Sample size** | **Male (%)** | **Age (years)** | **WC cut-off (cm)** | | **TG cut-off (mmol/L)** | | **Disease outcomes** | **Quality** |
| --- | --- | --- | --- | --- | --- | --- | --- | --- | --- | --- | --- |
|  |  |  |  |  |  | **M** | **F** | **M** | **F** |  |  |
| Ramezankhani, 2017 ^6, †^ | Iran | a. Cross-sectional study  b. Cohort study (12.4 years) | a. 12,012  b. 8,225 | a. 43.8  b. 45.1 | Male: 43.6±15.6  Female: 41.3±14.3 | 90 | 85 | 2.0 | 2.0 | CKD was defined as an eGFR of less than 60 ml/min per 1.73 m^2^. | 6 |
| Yu, 2018 ^7, ‡^ | a. Australia  b. China | Cross-sectional study | a. 1,454  b. 5,756 | a. 43.7  b. 43.7 | a. ≥25  b. ≥20 | 90 | 85 | 2.0 | 2.0 | Abnormal renal function was defined as eGFR <60 mL/min/1.73 m^2^. | 4 |
| Zhou, 2018 ^8^ | China | Cross-sectional study | 1,172 | 33.4 | Not reported | 90 | 85 | 2.0 | 2.0 | CKD was defined as an eGFR of less than 60 ml/min per 1.73 m^2^ or albuminuria (urinary albumin-to-creatinine ratio higher than 30 mg/g). | 6 |
| Qiu, 2020 ^9^ | China | Cross-sectional study | 31,296 | 40.6 | 55.64±11.35 | 90 | 80 | 1.7 | 1.7 | Decreased eGFR was defined as an eGFR value below 60 mL/min/1.73 m^2^. | 5 |
| Su, 2020 ^10^ | China | Cross-sectional study | 40,674 | 29.8 | > 40 | 90 | 85 | 2.0 | 1.5 | Urinary albumin-to-creatinine ratio higher than 30 mg/g. | 5 |
| Chen, 2022 ^*^ | China | Cohort study  (4 years) | 7,406 | 45.3 | 59.0±8.9 | 90 | 85 | 1.7 | 1.7 | CKD was defined as an eGFR of less than 60 ml/min per 1.73 m^2^ and/or self-reported physician diagnosed CKD. | 7 |

WC, waist circumference; TG, triglycerides; HTGW, hypertriglyceridemic-waist; M, male; F, female; CKD, chronic kidney disease; eGFR, estimated glomerular filtration rate.

^†^ Only data from the cross-sectional analysis (a) were included into the meta-analysis.

^‡^ Three different definitions were used for enlarged WC and high TG. Only the results with the most widely used definition (WC ≥90 cm and TG ≥2.0 mmol/L for men; WC ≥85 cm and TG for women ≥2.0 mmol/L) were included into the meta-analysis.

^*^ Data from current study.

**Supplementary Table S3. Summary of covariates and risk estimates of the studies included in meta-analysis**

| **Author, year** | **Covariates in main multivariate model** | **OR/HR (95%CI) for CKD in main multivariate model** | | |
| --- | --- | --- | --- | --- |
|  |  | **NTGW** | **HTNW** | **HTGW** |
| Li, 2014 ^1^ | Adjusted for age, sex, history of hypertension, history of coronary heart disease, history of stroke, history of malignancy, current smoker, current alcohol use, physical inactivity, educational status, diabetes, and hypertension. | OR^§^: 1.48 (1.01-2.16) | | OR: 2.09 (1.26-3.45) |
| Huang, 2015 ^2^ | Adjusted for age, history of coronary heart disease, history of stroke, history of malignancy, current smoker, current alcohol use, physical inactivity, educational status, diabetes, and hypertension. | OR^§^ (male): 1.14 (0.65-2.02)  OR^§^ (female): 1.08 (0.64-1.80) | | OR (male): 1.50 (0.81-2.78)  OR (female): 1.88 (1.05-3.36) |
| Zhang, 2015 ^3^ | Adjusted for age, gender, duration of diabetes, blood pressure, and HbA1c. | NA | NA | OR: 1.24 (1.03-1.50) |
| Zeng, 2016 ^4^ | Adjusted by age, sex, education, marital status, physical exercise, smoking, drinking, and family history of CVD, diabetes and hypertension. | OR (total): 1.38 (0.94-2.03)  OR (male): 1.73 (1.02-2.92)  OR (female): 1.06 (0.60-1.89) | OR (total): 0.39 (0.15-1.01)  OR (male): 0.59 (0.17-2.06)  OR (female): 0.24 (0.05-1.07) | OR (total): 1.95 (1.32-2.88)  OR (male): 1.88 (1.04-3.39)  OR (female): 1.66 (0.94-2.94) |
| Ma, 2017 ^5^ | Adjusted for sex, age, BMI, hypertension, history of diabetes, and glycosylated HbA1c. | OR: 1.61 (0.74-3.52) | OR: 1.90 (0.72-5.00) | OR: 2.81 (1.36–5.80) |

**Supplementary Table S3. Summary of covariates and estimated effects of the studies included in meta-analysis (continue)**

| **Author, year** | **Covariates in main multivariate model** | **OR/HR (95%CI) for CKD in main multivariate model** | | |
| --- | --- | --- | --- | --- |
|  |  | **NTGW** | **HTNW** | **HTGW** |
| Ramezankhani, 2017 ^6, †^ | Adjusted for age, smoking statues, education level, marital status, family history of diabetes, BMI, TC, diabetes and hypertension in women; adjusted for age, smoking statues, education level, marital status, physical activity level, BMI, TC, diabetes and hypertension in men. | a. OR (male): 1.02 (0.72-1.44)  OR (female): 1.44 (1.07-1.94)  b. HR (male): 0.88 (0.69-1.13)  HR (female): 0.99 (0.83-1.19) | a. OR (male): 1.04 (0.72-1.50)  OR (female): 1.68 (1.15-2.47)  b. HR (male): 0.98 (0.78-1.24)  HR (female): 1.06 (0.83-1.36) | a. OR (male): 0.77 (0.53-1.11)  OR (female): 1.37 (1.01-1.86)  b. HR (male): 0.86 (0.66-1.12)  HR (female): 1.07 (0.88-1.30) |
| Yu, 2018 ^7, ‡^ | Adjusted for age, gender, and BMI. | NA | NA | a. Definition 1: OR: 1.41 (0.64-3.10)  Definition 2: OR: 1.60 (0.71-3.64)  Definition 3: OR: 1.60 (0.61-4.18)  b. Definition 1: OR: 1.20 (0.86-1.66)  Definition 2: OR: 1.46 (1.01-2.09)  Definition 3: OR: 1.23 (0.81-1.87) |
| Zhou, 2018 ^8^ | Adjusted for sex, age, history of stroke, history of coronary heart disease, current smoker, current alcohol use, physical inactivity, education attainment, C-reactive protein, serum uric acid, BMI, diabetes and hypertension. | OR^§^: 1.52 (0.91-2.53) | | OR: 2.73 (1.13-6.62) |

| **Author, year** | **Covariates in main multivariate model** | **OR/HR (95%CI) for CKD in main multivariate model** | | |
| --- | --- | --- | --- | --- |
|  |  | **NTGW** | **HTNW** | **HTGW** |
| Qiu, 2020 ^9^ | Adjusted for age, sex, marital status, educational level, working status, smoking status, alcohol consumption, physical activity, BMI, hypertension, LDL-c, HDL-c, TC, and diabetes. | OR (total): 1.75 (1.41-2.18)  OR (male): 1.66 (1.19-2.33)  OR (female): 1.83 (1.36-2.45) | OR (total): 1.29 (0.99-1.68)  OR (male): 1.30 (0.92-1.85)  OR (female): 1.21 (0.80-1.85) | OR (total): 1.99 (1.54–2.58)  OR (male): 1.76 (1.17-2.64)  OR (female): 2.27 (1.61-3.20) |
| Su, 2020 ^10^ | Adjusted for age, sex, centers, education status, smoking habits, drinking habits, CVD status, diabetes history, hypertension history, use of diabetes or hypertension drugs status, BMI, eGFR, HDL-c, LDL-c, TC, AST, ALT, FBG, PBG, SBP, and DBP. | OR (total): 1.026 (0.898-1.172)  OR (male): 1.292 (0.945-1.767)  OR (female): 1.020 (0.878-1.186) | OR (total): 1.243 (1.096-1.410)  OR (male): 1.272 (0.986-1.639)  OR (female): 1.182 (1.016-1.376) | OR (total): 1.303 (1.132-1.499)  OR (male): 1.406 (1.057-1.870)  OR (female): 1.268 (1.074-1.496) |
| Chen, 2022 ^*^ | Adjusted for baseline age, sex, residence, education level, smoking and drinking status, BMI, HDL-c, history of DM, hypertension, and CVD, use of hypoglycemic agents, anti-hypertensive agents, and lipid-regulating agents, as well as baseline eGFR levels | OR (total): 1.48 (1.13-1.94)  OR (male): 1.57 (1.05-2.36)  OR (female): 1.45 (1.01-2.10) | OR (total): 1.16 (0.83-1.63)  OR (male): 1.13 (0.73-1.76)  OR (female): 1.25 (0.75-2.10) | OR (total): 1.82 (1.32-2.51)  OR (male): 1.53 (0.91-2.56)  OR (female): 2.15 (1.39-3.32) |

**Supplementary Table S3. Summary of covariates and estimated effects of the studies included in meta-analysis (continue)**

OR, odds ratio; HR, hazard ratio; WC, waist circumference; TG, triglycerides; NTGW, normal waist circumference with triglycerides phenotype; HTNW, normal waist circumference and high triglycerides phenotype; HTGW, hypertriglyceridemic-waist; CKD, chronic kidney disease; BMI, body mass index; SBP, systolic blood pressure; DBP, diastolic blood pressure; HDL-c, high-density lipoprotein cholesterol; LDL-c, low-density lipoprotein cholesterol; eGFR, estimated glomerular filtration rate; CVD, cardiovascular disease; ALT, alanine aminotransferase; AST, aspartate aminotransferase; FBG, fasting blood glucose; PBG, 2 h post-load blood glucose; HbA1c, glycated hemoglobin.

^†^ Only data from the cross-sectional analysis (a) were included into the meta-analysis.

^‡^ Three different definitions were used for enlarged WC and high TG. (Definition 1: WC ≥85 cm and TG ≥1.5 mmol/L for both men and women. Definition 2: WC ≥90 cm and TG ≥2.0 mmol/L for men; WC ≥85 cm and TG ≥1.5 mmol/L for women. Definition 3: WC ≥90 cm and TG ≥2.0 mmol/L for men; WC ≥85 cm and TG for women ≥2.0 mmol/L). Only the results with the definition 3 were included into the meta-analysis.

^§^ NTGW and HTNW phenotypes were combined into one group.

^*^ Data from current study.

**
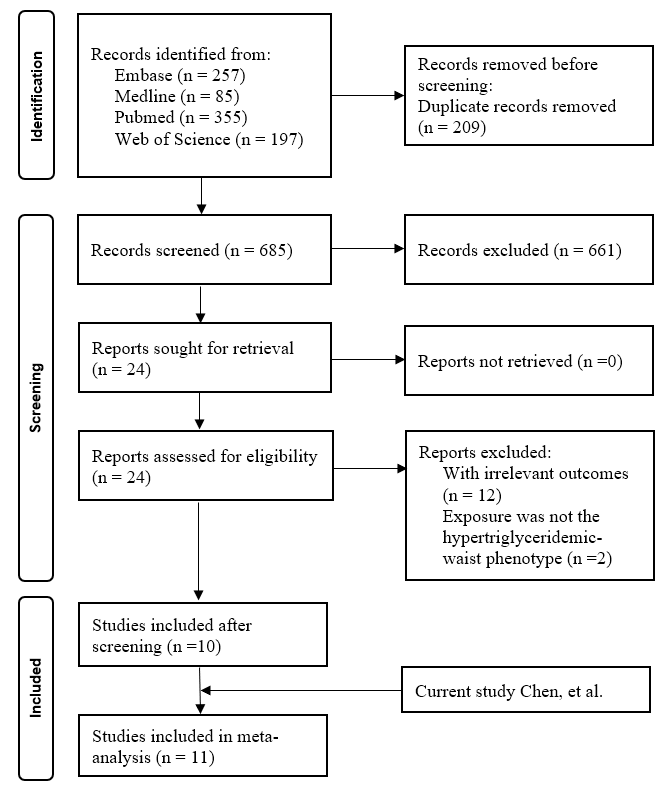
Supplementary Fig S1. PRISMA flow diagram of the study selection process**

**
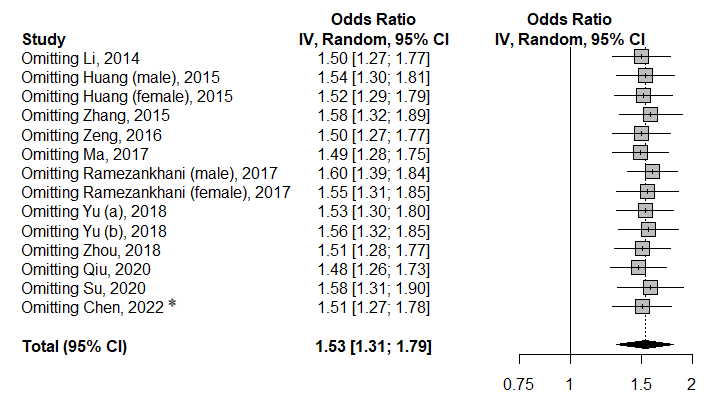
Supplementary Fig S2.** **Examining the influence of individual study by leave-one-out method**

*** Data from current study**

**
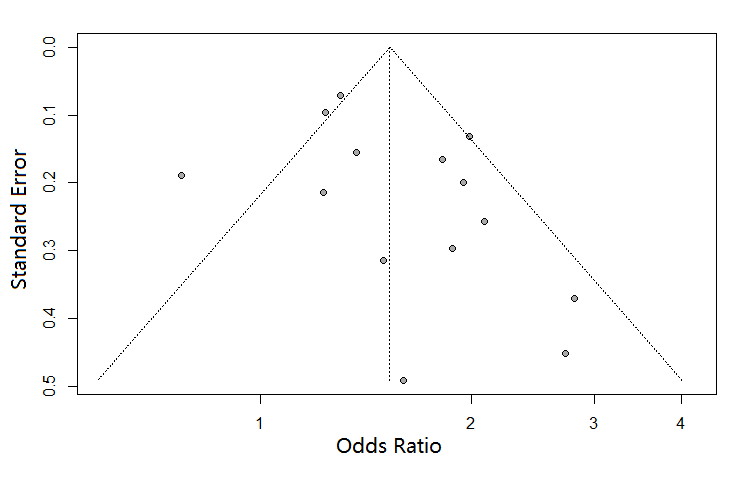
** **Supplementary Fig S3. Funnel plot of included studies**

**
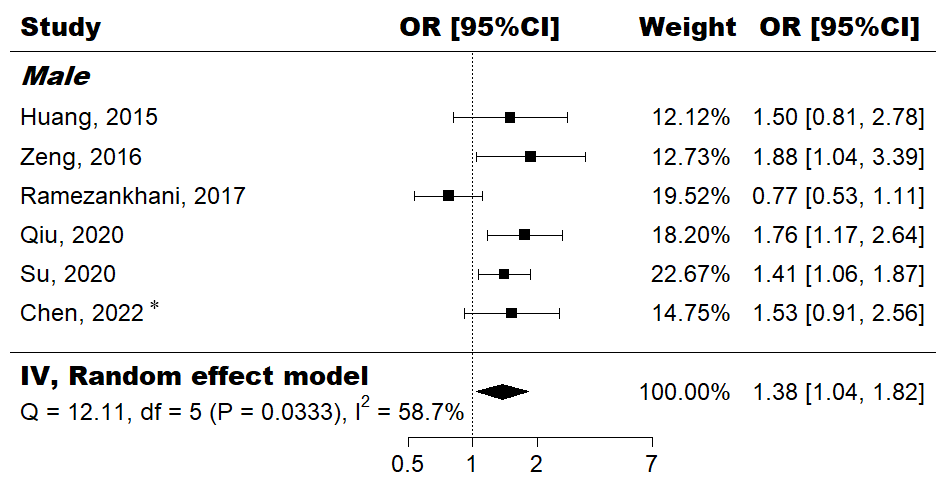
Supplementary Fig S4. Forest plot summarizing the association between hypertriglyceridemic-waist phenotype and chronic kidney disease in males**

*** Data from current study**

**
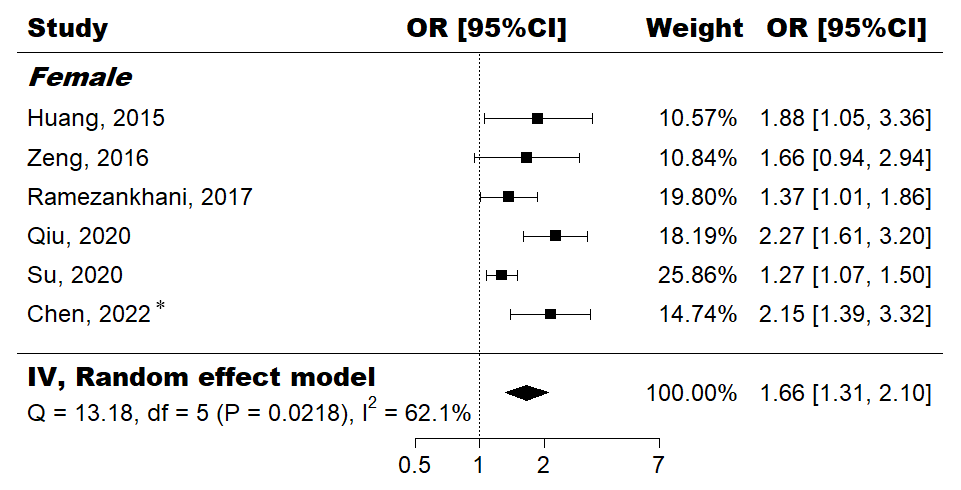
Supplementary Fig S5. Forest plot summarizing the association between hypertriglyceridemic-waist phenotype and chronic kidney disease in females**

*** Data from current study**

**Reference**

1 Li, Y. *et al.* Hypertriglyceridemic waist phenotype and chronic kidney disease in a Chinese population aged 40 years and older. *PLoS One* **9**, e92322, doi:10.1371/journal.pone.0092322 (2014).

2 Huang, J. *et al.* Visceral adiposity index, hypertriglyceridemic waist phenotype and chronic kidney disease in a southern Chinese population: a cross-sectional study. *Int Urol Nephrol* **47**, 1387-1396, doi:10.1007/s11255-015-1040-y (2015).

3 Zhang, L. *et al.* The hypertriglyceridaemic-waist phenotype in relation to microalbuminuria in patients with type 2 diabetes. *Diabetes* **64**, A613, doi:10.2337/db1523742461 (2015).

4 Zeng, J. *et al.* The Association of Hypertriglyceridemic Waist Phenotype with Chronic Kidney Disease and Its Sex Difference: A Cross-Sectional Study in an Urban Chinese Elderly Population. *Int J Environ Res Public Health* **13**, doi:10.3390/ijerph13121233 (2016).

5 Ma, C.-M. *et al.* The Relationship between Hypertriglyceridemic Waist Phenotype and Early Diabetic Nephropathy in Type 2 Diabetes. *Cardiorenal Med* **7**, 295-300, doi:10.1159/000477828 (2017).

6 Ramezankhani, A., Azizi, F., Ghanbarian, A., Parizadeh, D. & Hadaegh, F. The hypertriglyceridemic waist and waist-to-height ratio phenotypes and chronic kidney disease: Cross-sectional and prospective investigations. *Obes Res Clin Pract* **11**, 585-596, doi:10.1016/j.orcp.2016.11.003 (2017).

7 Yu, D. *et al.* Hypertriglyceridemic-waist is more predictive of abnormal liver and renal function in an Australian population than a Chinese population. *Obes Res Clin Pract* **12**, 438-444, doi:10.1016/j.orcp.2018.07.010 (2018).

8 Zhou, C., Li, Y., Shao, X. & Zou, H. Identification of chronic kidney disease risk in relatively lean Southern Chinese: the hypertriglyceridemic waist phenotype vs. anthropometric indexes. *Eat Weight Disord* **23**, 885-892, doi:10.1007/s40519-017-0476-8 (2018).

9 Qiu, Y. *et al.* Association of hypertriglyceridemic waist phenotype with renal function impairment: a cross-sectional study in a population of Chinese adults. *Nutr Metab (Lond)* **17**, 63, doi:10.1186/s12986-020-00483-7 (2020).

10 Su, W., Wang, J. & Mu, Y. Association Between Hypertriglyceridemic Waist Phenotype and Increased Urinary Albumin-Creatinine Ratio in Chinese Adults: The REACTION Study. *Diabetes Metab Syndr Obes* **13**, 2965-2974, doi:10.2147/DMSO.S257736 (2020).
